# Bringing Persons With Dementia to Health Care Visits: Caregiver Perspectives

**DOI:** 10.1093/geroni/igaf122.911

**Published:** 2025-12-31

**Authors:** Megan Gately, Jaye McLaren, Lauren Moo

**Affiliations:** VA Bedford Health Care System, Bedford, Massachusetts, United States; VA Bedford Healthcare System, Bedford, Massachusetts, United States; VA Bedford Healthcare System, Bedford, Massachusetts, United States

## Abstract

Caregivers of persons living with dementia (PLWD) face many care challenges including the need to provide increasing support to PLWD day-to-day needs as the disease progresses. Caregivers’ management of PLWD health care is an acknowledged aspect of their role, and includes scheduling appointments, managing medications, and communicating with clinicians. Yet little is known about the full scope of that support particularly in the realm of attending health care visits with PLWD. This study presents factors associated with attending health care visits with PLWD. Factors were identified by caregivers of PLWD residing in the community during semi-structured qualitative interviews (N = 24). Content analysis of interview data revealed three categories related to the experience of attending health care visits with PLWD: 1) Preparing for the Visit, 2) Visit-Related Time and Travel, and, 3) In-visit Experience, highlighting associated barriers and facilitators within each category. Barrier statements generally reflected the negative impact of cognitive impairment, such as PLWD anxiety, confusion, and repetitive questioning about the purpose of the visit, at times well in advance of the visit. Facilitators statements generally reflected the benefit of attending visits as opportunities for social participation and providing caregivers an opportunity to engage with care teams. Taken together, these factors elucidate the experience of taking PLWD to health care visits from the perspectives of caregivers, highlighting opportunities to mitigate more negative aspects of that experience.

